# A new journal... on spindle cells

**DOI:** 10.1186/2045-3329-1-1

**Published:** 2011-07-25

**Authors:** Paolo G Casali, Angelo P Dei Tos

**Affiliations:** 1 Istituto Nazionale Tumori, Via G. Venezian 1, Milano, 20133, Italy; 2 General Hospital of Treviso, Piazza Ospedale 1, Treviso, 31100, Italy

## Abstract

Welcome to *Clinical Sarcoma Research *a new open access, online medical journal providing a forum for clinical knowledge on rare solid cancers - sarcomas. We believe there is a vacuum, which this effort may hope to fill at least in part. Indeed, we ought to share first-hand medical experience and clinically meaningful translational ideas much more within the sarcoma community worldwide. This journal is intended to be one of the many tools we need for this purpose.

## Networking is the keyword when dealing with rare diseases, including rare cancers

*Clinical Sarcoma Research *was conceived by two "networks of excellence" focusing on soft tissue and bone sarcomas, namely CONTICANET and EUROBONET, which were initially funded by the European Commission within its 6^th ^Framework Programme for Research and Technological Development. At the time this open-access journal was founded, towards the end of their EU-funded life span, these networks were considering merging and becoming one entity. Now, a new European Commission-funded initiative called EUROSARC will take off in 2012, thereby continuing and expanding the research activity generated within both CONTICANET and EUROBONET.

CONTICANET and EUROBONET were originally intended to foster "excellence" in clinical and translational research, but they also inevitably focused on quality of care (e.g., by dealing with clinical practice guidelines, and so forth). Nothing, in fact, can be done in rare diseases by separating care and research.

In rare cancers, even a single clinical case can teach a lot. Is it care or research? Occasionally, a single patient may lead translational scientists to conceive new ideas, by serendipity. And, vice versa, a single case can be sufficient to provide convincing proof of a strong translational hypothesis. In the recent past this paradigm allowed the sarcoma community to make big steps forward. It is intuitive that rare cancers can by no means be less complex than frequent ones. However, in rare cancers we cannot afford the same quality of evidence generated by big numbers. We would need dissemination tools to accommodate for this. Sometimes, medical decisions are made in rare cancers by generously sharing personal experience via e-mail within narrow, world-spanning medical circles. Why not make all this public? Sometimes, in rare cancer patients, new agents are used off-label or on a compassionate basis. Why not make these cases public, even when the outcome is negative? This would help limit the publication bias, which is the real biasing factor of published anecdotal evidence. At times, a preliminary translational finding could have led to clinical results had it been made known in a timely manner. Why not publish it earlier? Sometimes, expert reviews can prove precious for clinicians occasionally dealing with a very rare cancer patient, or commentaries may stimulate innovative thinking in highly dedicated communities. Why not publish them, even if they focus on very narrow topics, of little interest to many, and/or they collect anecdotal evidence rather than systematic reviews?

This is the background for *Clinical Sarcoma Research*. By giving preference to highly selected subgroups within this family of rare cancers, this journal will accept:

1. clinical studies, including small ones and/or ones reporting negative results;

2. case reports and small case series analyses;

3. reports of clinical or research efforts methodologically pursuing innovative ways to generate new evidence;

4. review papers on highly specific topics;

**5. commentaries designed to stimulate discussion and innovative thinking in the sarcoma community**.

This will be an open-access journal, exploiting all benefits offered by such a powerful instrument. Thus, published items will be freely accessible to all readers, their copyright will be retained by the authors and they will be deposited in PubMed Central and other full-text repositories. A widely accessible journal of this type may become a key resource for the worldwide sarcoma community, filling some of the gaps in medical literature that could limit knowledge-sharing on the diseases this community is concerned with.

We will be especially keen to consider papers which could be rejected by other medical journals not because of their low quality, but because of their low interest to a general medical audience and/or of methodological constraints directly related to the rarity of sarcomas. We would be happy for this journal to become a tool for courageous attempts to innovate the methodology of knowledge-building in rare diseases. We will be open to Bayesian statistics, which look especially promising when numbers are low. Possibly, instruments other than medical journals will be used in the future to share anecdotal evidence in real time, such as web-based tools. However, medical journals will still perform the pivotal role of providing peer-reviewed literature of a trusted quality and may also summarise the evidence, and/or host expert discussions about it. This journal may well serve the new dissemination requirements of innovative ways to develop new therapies in sarcomas, as a model, perhaps, for other rare, and less rare, diseases.

Today, a formidable number of large clinical trials on new targeted agents in cancer are published in prestigious journals. They narrow uncertainty through huge numbers, but this often comes at the price of narrowing differences in survival, or surrogates thereof. Just because of the need for such huge numbers, relevant subgroups (i.e. possible targets) may be overlooked, or not looked for, in spite of the targeted nature of investigated drugs. Herein, we will focus on a family of rare cancers and encourage to further focus on their subgroups. We welcome the plausibility of major outcomes, even if at the price of an excess of uncertainty. We will welcome clinical precision, even if at the price of less statistical power. Our argument is that rare cancer patients would be discriminated against, if the same volume of evidence is required as for diseases with much higher numbers.

Thank you for joining us and contributing to the good cause of rare solid cancers.

Paolo G. Casali

Angelo Paolo Dei Tos

Editors-in-Chief *- Clinical Sarcoma Research*

